# Oncolytic Sendai Virus Therapy of Canine Mast Cell Tumors (A Pilot Study)

**DOI:** 10.3389/fvets.2018.00116

**Published:** 2018-06-04

**Authors:** Galina V. Ilyinskaya, Elena V. Mukhina, Alesya V. Soboleva, Olga V. Matveeva, Peter M. Chumakov

**Affiliations:** ^1^Engelhardt Institute of Molecular Biology, Moscow, Russia; ^2^Blokhin Cancer Research Center, Moscow, Russia; ^3^Veterinary Clinic of Herzen Oncology Research Institute, Moscow, Russia; ^4^Chumakov Federal Scientific Center for Research and Development of Immune-and-Biological Products, Moscow, Russia; ^5^Biopolymer Design LLC, Acton, MA, United States

**Keywords:** canine mastocytoma, mast cell tumor, MCT, oncolytic virus, Sendai virus, virotherapy

## Abstract

**Background:** Canine mastocytomas (mast cell tumors) represent a common malignancy among many dog breeds. A typical treatment strategy for canine mastocytomas includes surgery, chemo- and radio-therapy, although in many cases the therapy fails and the disease progression resumes. New treatment approaches are needed.

**Aims:** The goal of this pilot study was to examine safety and efficacy of oncolytic Sendai virus therapy administered to canine patients with cutaneous or subcutaneous mastocytomas.

**Materials and Methods:** Six canine patients, with variable grades and stages of the disease, received virus therapy, either as a monotherapy, or in combination with surgery. The therapy included two or more virus applications administered weekly or biweekly. Each application of Sendai virus (10^7^-10^8.6^ EID50) consisted of multiple individual 0.01–0.1 ml injections delivered intratumorally, intradermally around a tumor, and under a tumor bed.

**Results:** The treatment was well tolerated, with minor transitory side effects. Of the six dogs, two did not receive surgery or any other treatment besides the virus injections. The other four animals underwent radical or debulking surgeries, and in three of them the subsequent administration of Sendai virus completely cleared locally recurrent or/and remaining tumor masses. Five dogs demonstrated a complete response to the treatment, the animals remained disease free during the time of observation (2–3 years). One dog responded only partially to the virotherapy; its after-surgical recurrent tumor and some, but not all, metastases were cleared. This dog had the most advanced stage of the disease with multiple enlarged lymph nodes and cutaneous metastases.

**Conclusion:** The results of the pilot study suggest that Sendai virus injections could be safe and efficient for the treatment of dogs affected by mastocytomas.They also suggest the need of further studies for finding optimal schemes and schedules for this kind of therapy.

## Introduction

Mast cell tumors (MCTs) are common canine malignancies representing up to 20% of canine skin cancers. Usually they are diagnosed by fine needle aspiration (1–3). A treatment strategy for these tumors represents a challenge because MCT response to a treatment is highly variable and the advanced disease is frequently lethal (2, 3). The age distribution of MCT onset peaks from 6 to 8 years. Frequency of occurrence varies between breeds. Beagles, bulldogs, Boxers, Boston terriers, Rhodesian ridgebacks, pugs, Weimaraners, Labradors, and golden retrievers develop MCTs most frequently (4–6). Some breeds, such as Pugs, tend to develop less aggressive and more differentiated tumors (7). In contrast, Labrador retrievers and golden retrievers develop more aggressive mastocytomas (3). Appearance of these tumors is highly variable; they might be represented by raised or very deep lesions. Some dogs develop solitary and some multiple lesions. The multiple lesions might be represented by related or unrelated MCTs (1–3).

Normal mast cells, which represent a subtype of white blood cells, have multiple roles in immune system functioning. They are involved in defense against pathogens and in wound healing. Mast cells derive from pluripotent CD34+ hematopoietic stem cells that reside in bone marrow (8). Mast cells are located in connective tissue and, when activated, rapidly release the contents of their granules—histamine, proteases, cytokines and heparin—into the interstitium contributing to the inflammation response (9). Dysfunctional release of the contents of MCT's granules produces significant secondary damage such as gastric ulcers, internal bleeding, and a range of allergic reactions (10).

The outcome of MCT disease depends on many factors. Survival time is linked to MCT's grade, stage, the location of the primary tumor, status of surgical margins, and mitotic index (number of mitotic figures per 10 high-power fields) (11–15). The following criteria are employed for MCT's grade assignment; type of cell arrangement, shape of cells, presence of ample cytoplasm and cytoplasmic boundaries, shape of nuclei, type, and size of intra-cytoplasmic granules, presence of giant and multinucleated cells, mitotic index, and presence of edema, necrosis and hemorrhage (16, 17). The most common grading system for cutaneous MCT developed by Patnaik et al. (16) defines grade 1 as a tumor formed by well-differentiated, grade 2—by an intermediate, and grade 3—by poorly differentiated mast cells. The disease grade correlates with survival rate. After a surgery 93% of animals with grade 1 MCT survived at least 4 years, however only 44% of animals with MCT grade 2 and 6% with MCT grade 3 survived that long (16).

Kiupel system of cutaneous MCT grading is more recent, more precise and achieves better agreement among veterinary pathologists. Moreover, it more accurately predicts MCT's biological behavior. The system defines only two histological categories, high-grade and low-grade. High-grade tumors were related to shorter survival time, on average <4 months. In contrast, for low-grade MCT average survival time was more than 2 years. Such features as mitotic index of 7 or more, presence of 3 or more multinucleated cells, presence of 3 or more bizarre nuclei, or detection of karyomegaly in 10 high power fields define high-grade tumors (17). Kiupel MCT grading system is more specific and clear cut, yet many veterinary pathologists prefer using Patnaik grading system. Patnaik system was used in the present study.

Other factors such as intratumoral blood vessel density (18), neutrophil to lymphocyte ratio (19), and leukocyte profile and count (20) were also shown to play a role in survival prognosis. Analysis of genomic alterations has revealed some useful prognostic markers, such as deletions within PTEN and FAS and amplifications of MAPK3, WNT5B, FGF, FOXM1, and RAD51 genes, which were associated with shorter survival times (21). Also, certain haplotypes harboring the hyaluronidase genes were identified as disease associated risk factors (22). Finally, a connection was found between c-KIT gene mutations, elevated c-KIT mRNA expression and a higher grade of tumor (15, 23–27). Consequently, c-KIT mutations in tumor tissues are related to shorter survival times (28, 29). These mutations are present in 50% of high grade and in 9–15% of overall MCT cases (28–30).

Surgery is an effective treatment for well- or intermediate-differentiated tumors, but only if the disease did not spread from the original location. In dogs subjected to surgery alone, margins status and tumor grade predict the disease outcome. As expected, infiltrated margins and high tumor grade (according to Kiupel system) were related to higher reoccurrence rate (31). Tumor-free margins of 3 mm were shown to be sufficient to prevent reoccurrence of low-grade tumors (13). However, high-grade tumors reoccurred frequently (50% or more), despite the width of their tumor-free margins (16, 32).

There is no standard protocol for MCT treatment and the only FDA-approved drug is Toceranib (Palladia) (33). However, different treatment options that include radiation and variable chemotherapeutic protocols have been established in many veterinary facilities. Relevant publications with treatment side effects and efficacy results are listed in Table 1. Many treatment protocols provide significant life extension for canine patients. However, the treatment failure rate for advanced MCTs is high and none of the large-scale clinical trials have achieved long-lasting complete response rates exceeding 15%. Radio- and cytostatic chemotherapy are less effective against tumor cells that divide slowly, and the absence of c-KIT mutations makes the tumors less likely to respond to protein kinase inhibitors (33, 45). So, tumors with comparatively low mitotic index or without c-KIT mutations, which represent up to 80% of MCT malignancies (12), are much less treatable by chemotherapy or radiation.

**Table 1:** Outcomes of clinical testing of varying MCT treatment protocols.

***N***	**Name of chemical compound**	**Number of animals**	**Type of disease**	**Side effects, grade, percent of affected animals (if reported)**	**References**
1	Lomustine (CCNU), (cell cycle neutral)	19	Grades 1–3	Neutropenia	(34)
	Eight dogs had a measurable response. One dog had a durable complete response for 440 days. Seven dogs had a partial response for a median duration of 77 days. Six dogs had stable disease for a median duration of 78 days.				
2	Lomustine	81	Non-resectable, grades 2 or 3	Neutropenia or gastrointestinal effects, grades 3 and 4 (86%), hepatopathy (33%)	(35)
	One dog showed partial response. Seven dogs had stable disease at the end of the study.				
3	Lomustine combined with prednisone	12	Incompletely excised, grade 2	Two fatal liver failures	(36)
	Seven dogs survived 2 years without disease progression. Two died from liver failure, indicating substantial liver toxicity of the two-drug combination.				
4	Surgical cytoreduction, radiation	31	Incompletely excised, grade 3	Not reported	(37)
	Eleven dogs were alive at least 1 year without evidence of disease progression. Seventeen dogs died or were euthanized because of disease progression. The median disease free survival was 839 days. Median duration of overall survival was 845 days. Dogs with tumors ≤ 3 cm maximum diameter before surgery survived longer than dogs with tumors >3 cm. The remission rate was 65% and survival rate was 71% at 1 year after treatment.				
5	Surgical cytoreduction, radiation, prednisone	19	Cutaneous MCT, stage 2	Not reported	(38)
	The median disease-free survival was 1,240 days.				
6	Surgery, radiation, prednisone/vinblastine	61	High risk for metastasis	Neutropenia, grades 3–4 (6.5%)	(39)
	Twenty-four dogs developed disease progression. The median progression-free interval was 1,305 days, and the median overall survival was not reached. Sixty-five percent of all animals were alive at 3 years, but all dogs with grade 2 disease were alive at 3 years. The median overall survival for dogs with grade 3 disease was 1,374 days.				
7	Surgical cytoreduction, radiation, vinblastine, lomustine, and prednisone	21	Grade 2, stage 2	Neutropenia, hepatotoxicity, grade 2–3 toxicities (63%)	(40)
	Dogs that were treated with radiation, surgery, and chemotherapy had longer median overall survival than those that were treated only with surgery and chemotherapy (2,056 days vs. 1,103, respectively).				
8	Vinblastine, lomustine, and prednisone	17	Non-resectable, grades 2 and 3	Hepatotoxicity (9%)	(41)
	Five showed a complete response and six a partial response. The median progression-free survival time among treated dogs was 489 days.				
9	Vinorelbine	24	Previously untreated cutaneous MCT	Neutropenia and gastrointestinal toxicity, grade 1-3	(42)
	One dog achieved complete and two partial responses. The response was measured after only 1 week and only after single dose of the drug injection.				
10	Prednosolone, chlorambucil	21	Non-resectable, grades 2 and 3	Not reported	(43)
	Three dogs achieved a complete response and five partial responses. The median progression-free interval for the eight responders was 533 days, and the median survival time for all dogs in the study was 140 days.				
11	Paclitaxel (Paccal Vet)	29	Non-resectable, grades 2 or 3	Neutropenia, grades 3–4 and leucopenia, grades 1–2	(44)
	Complete or partial responses were observed in more than half of the animals. The median time to progression in treated animals was 247 days.				
12	Paclitaxel	168	Advanced stage, non-resectable, grades 2 or 3	Neutropenia or gastrointestinal effects, grades 3 and 4 (73%)	(35)
	Three percent of dogs showed complete and ~60% partial response or stable disease at the end of the study. Paclitaxel treated dogs were 6.5 times more likely than lomustine treated dogs to respond to chemotherapy.				
13	Mastinib	200	Non-metastatic recurrent or non-resectable, grades 2 or 3	Gastrointestinal toxicity, grades 1 and 2 (36%)	(45)
	Treatment for 168 days significantly increased median time to disease progression (75 days in the placebo group and 118 days in the treatment group). The overall median survival time was 340 days in the placebo group and 491 days in the treatment group. The difference between placebo and treatment groups in median survival time duration reached significance only among the dogs with tumors expressing a mutant form of the c-KIT gene (182 vs. 417 days). For the entire population of dogs this difference was not significant.				
14	Mastinib	132	Non-resectable, grades 2 or 3	Gastrointestinal toxicity, grades 2 and 3	(46)
	Complete responses were observed in 6 out of 67 treated dogs. Treatment for two years significantly increased median survival time (322 days for the placebo group and 617 days in the treatment group).				
15	Toceranib (Palladia), FDA approved	145	Reoccurred MCT	Gastrointestinal effects, grades 3 and 4 (34 %)	(33)
	Animals received medication every other day for 6 weeks. Twenty-one dogs achieved complete and 41 partial response. Among the 62 responders, the median duration of the objective response and time to tumor progression were 84 and 126 days, respectively.				

Oncolytic virotherapy is an emerging therapeutic option for cancer treatment. However, there are very few reports that describe its application in veterinary medicine. An adenovirus-based construct was tested for treatment of aggressive canine melanomas (47). One dog in this study was diagnosed with an advanced stage III oral melanoma. A combination of viral treatment and cytoreductive surgery caused complete tumor clearance and long term disease free survival of this dog. Another dog was diagnosed with conjunctival melanoma that progressed very rapidly. Multiple viral injections without surgery caused disease stabilization and long progression free survival of this dog (47). A different study with the same adenovirus construct introduced by a few intratumoral injections ended with complete responses in 5 out of 19 dogs affected by malignant melanomas (48). Three relatively recent reviews summarize results of oncolytic viruses-based therapeutic approaches for canine malignancies (49–51).

Oncolytic potentials of Sendai virus have been studied mainly in Japan. Sendai virus belongs to Paramyxoviridae family and its pathogenicity is host-restricted to rodents (52). It can produce transmittable respiratory tract infections in mouse colonies worldwide (52) and is completely safe for humans (53, 54).

A number of studies have demonstrated that an attenuated (through genetic engineering) Sendai virus loses its pathogenicity for rodents. However, it can spread in tumors and kill malignant cells, while sparing the surrounding normal cells. The virus was shown to suppress or completely eradicate a number of tested experimental tumors, including xenografted human tumors, including fibrosarcomas, neuroblastomas, pancreatic, colon, and prostate carcinomas (55–58). A number of human tumors in rat xenograft models also responded to Sendai virus therapy including melanomas, neuroblastomas, squamous cell, hepatocellular, and prostate carcinomas (59). Even after inactivation with ultraviolet light, Sendai virus particles were able to suppress or eradicate colon (60), bladder (61), and kidney (62) cancers in syngeneic murine models. Similar results were demonstrated for mouse xenografts of human prostate cancer (63).

The present report describes six canine MCT cases treated with Sendai virus strain attenuated through multiple passages in embryonated chicken eggs. In the study we used two different dosages of the virus in order to get information if doses with a lower titer would be able to clear the tumors, and if doses with a higher titer would cause unwanted side effects.

## Materials and methods

Protocols and procedures described in this study were reviewed and approved by the animal ethics committee of the Veterinary Clinic of Herzen Oncology Research Institute.

### Tumor grading and staging

Tumors were graded according to Patnaik et al. (16) by a single board-certified veterinary pathologist. The following criteria were used for grade assignment: cell arrangement; shape of cells and their nuclei; presence of cytoplasmic boundaries; type, quantity, and size of intra-cytoplasmic granules; mitotic index; and presence of multinucleated cells, edema, necrosis, and hemorrhage. Histological examination was not performed for cases 1 and 6 because the animals didn't undergo surgical treatment. So, the determination of tumor grade for these cases was based on cytology only and, consequently it was less precise in comparison with other cases. Tumors staging for case 1 and case 6 was not performed. Primary diagnosis was established by fine-needle aspiration technique, and if a surgery was performed, the diagnosis was a subject of confirmation by histological examination of the excised tumor. Mitotic index for histological examination was evaluated per 10 high-power (400 X) fields (HPF), field size 2.7 mm^2^. Secondary growth and lymph nodes were examined using fine-needle aspiration. Tumor staging was done by histological examination of excised tumor masses, by palpation and fine-needle aspiration of lymph nodes and visible metastases. The staging was not very precise because radiography, ultrasound, bone marrow, and organ biopsies were not performed.

### Prednisone treatment

To avoid any immunosuppressive effects prednisone was not routinely administered during Sendai virus therapy. However, one dog (case 3), which had the most advanced disease, was treated with regular daily oral prednisone (1 mg/kg) as palliative care during 28 days before euthanasia. Another dog (case 5) was treated once with injectable prednisone (2 mg/kg) after an mastocytoma degranulation event that was not related to the viral treatment.

### Viral preparation

Sendai virus was prepared as described (64) with some modifications. In brief, diluted virus seed (100 ul) was inoculated into allantoic cavity of 10-day embryonated eggs. Virus containing allantoic fluid was harvested after 3 days of incubation at 37°C. The virus titer was determined by infection of embryonated eggs with serial dilutions and expressed as 50% Egg Infective Dose/ml (EID50/ml). Sendai virus material used in animal experiments was manufactured by two different procedures that were performed in two different facilities. The first preparation was done in our research facility and included virus purification as well as concentration through ultra-filtration dialysis of collected allantoic fluid. The second virus preparation was purchased from Charles River Laboratory (North Franklin, CT 06254). It included Sendai virus-containing allantoic fluid without any purification that was directly lyophilized, and used upon rehydration.

### Virus purification by ultra-filtration dialysis

Virus-containing allantoic fluid was clarified using low-speed centrifugation (1,500 g for 10 min) and subjected to ultra-filtration with a Pellicon 2 cassette (Millipore, mini Ultra-filtration Module Biomax-C 0.1 m^2^ [P2B300C01]). The initial sample volume was reduced to 1/10, and then washed with 50 volumes of 0.14 M NaCl solution. The filtration dialysis aimed removing all material with molecular weight <300,000. After filtration the concentrated viral material was diluted to 10^7^ EID50/ml, aliquoted into cryotubes and stored at −80°C.

### Lyophilization of sendai virus in allantoic fluid without purification

Virus-containing allantoic fluid was subjected to lyophilization. For this purpose 50 ml of 5% stabilizing medium was mixed with 100 ml of allantoic fluid. The stabilizing medium consisted of 10 g sucrose (Sigma-Aldrich, S0389) and 5 g lactalbumin hydrolysate (Sigma Aldrich, CAS Number: 91079-44-6) per 100 ml. The mixture was aliquoted into 1.5 ml portions, placed into cryotubes, and subjected to lyophilization. This freeze drying procedure was carried out using a Christ alpha 1–4 LSC freeze-dryer (Martin Christ Freeze Dryers). After the lyophilization procedure and reconstitution in sterile water the virus titer was 10^8.6^ EID50/ml. The vials were stored at −20°C.

### Virus treatment

Virus treatments were administered at 2-week intervals for case 1 and 6 and 1-week intervals for the remaining cases. For each treatment up to ten 10–100 μl aliquots from 1 ml of virus preparation were injected into multiple sites intratumorally, intradermally around the tumor site and under the tumor bed. Injections containing virus purified by ultra-filtration with a titer of 10^7^ EID50/ml were administered in cases 1, 2, 3, and 5. The injections containing virus lyophilized directly from allantoic fluid with a titer of 10^8.6^ EID50/ml were administered in case 4. RECIST evaluation criteria were used for assessing responses of tumors to the viral treatment.

## Results

Six canine patients underwent oncolytic Sendai virus treatment. Virotherapy was administered either as a monotherapy, or in a combination with surgery. The treatment was well tolerated; minor transitory side effects included fever, fatigue, and pain at the site of viral injection. The side effects were classified as grade 1 according to common terminology criteria for adverse events of the Veterinary Cooperative Oncology Group 2 (65). A side effects summary is presented in Table 2. Virotherapy treatment efficacy results are described below.

**Table 2:** Summary of side effects that occurred during Sendai virus therapy.

**Side effect**	**Occurrence**
Pyrexia (20 h after the injection)	Cases 2 and 3
Asthenia	Cases 3 and 5
Edema at the site of injection	Case 4
Vomiting	0
Diarrhea	0
Pain or tenderness at site of injection	Case 3, 4, and 5
Musculoskeletal pain	0
Decreased appetite	Case 3

*All side effects were classified as grade 1 according to common terminology criteria for adverse events of the Veterinary Cooperative Oncology Group 2 (65)*.

### Case 1

**Figure 1:**
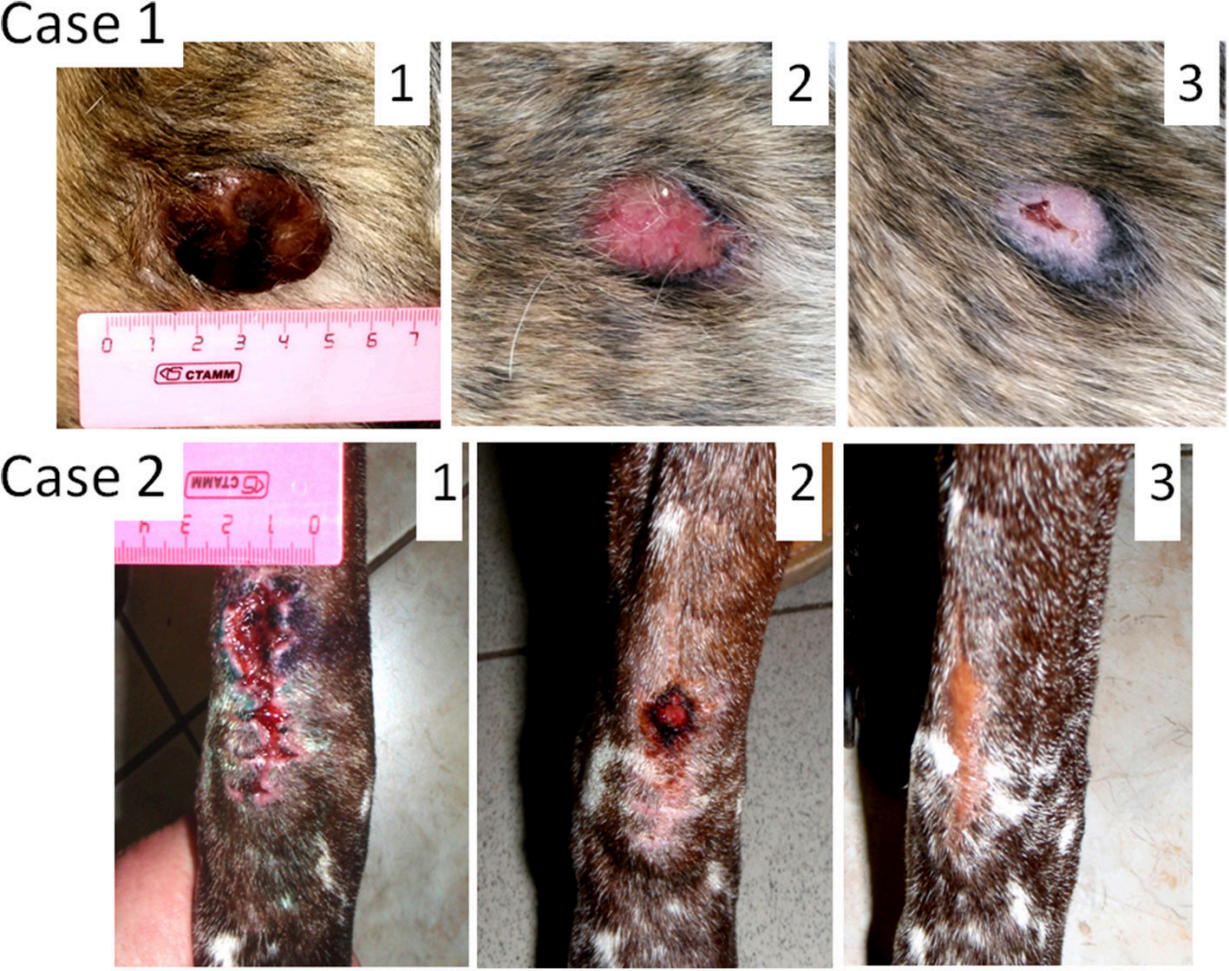
Oncolytic Sendai virus MCT treatments results (Cases 1 and 2). **Case 1**. Male dog of mixed breed of 7 years old was presented with cutaneous, ulcerated, and poorly differentiated mastocytoma (35 mm diameter) located near his anus. The tumor stage was not assigned. (1) Primary tumor before any treatment; (2) MCT site, 2 weeks after the first virus treatment; (3) MCT site, 4 weeks after the first virus treatment. **Case 2**. Male German shorthaired pointer of 9 years old was presented with subcutaneous, regional (stage 2) intermediately differentiated mastocytoma. The primary tumor was excised without clean margins (is not shown). (1) MCT secondary growth 1 week after the surgical procedure; (2) MCT site, 2 weeks after the first virus treatment; (3) MCT site, 5 weeks after the first virus treatment.

Male dog of mixed breed, 7 years old, presented with ulcerated cutaneous mass (35 mm in diameter) located near his anal gland (Figure 1, **Case 1**). According to the dog's owner the tumor grew to the size of 35 mm during 3 weeks period. Fine-needle aspiration revealed that the tumor was poorly differentiated. The tumor stage was not assigned. Because of the tumor size and its proximity to anus, a complete surgical excision with clear margins was not possible. The treatment option was to try oncolytic virotherapy for tumor clearance. Sendai virus in a dose of 1 ml 10^7^ EID50/ml was administered with 2-week intervals. The tumor was cleared after only two viral treatments; however two additional treatments were administered afterwards to ensure the effect. Three years after the treatment the dog does not have any signs of tumor recurrence. This dog was the first patient to receive the virus treatment at the Veterinary Clinic of the Herzen Oncology Research Institute. The absence of visible side effects in this dog encouraged more frequent (weekly) treatments for later canine patients.

### Case 2

Male German shorthaired pointer, 9 years old, presented with subcutaneous MCT located in the elbow region (Figure 1, **Case 2**). Fine-needle aspiration revealed an intermediately differentiated tumor. The tumor was excised without clean margins. The surgery has curative intent, but only tumor debulking was achieved. Histological examination of the excised tumor mass revealed a mitotic index of 5 (10 × HPF) and confirmed that the tumor was intermediately differentiated. It was also noticed that the tumor penetrated underlying muscle tissue and therefore was staged as regional (stage 2). As expected, multiple lesions likely of the same tumor grade (tested by fine-needle aspiration) appeared at the surgical site 2 weeks after the surgery. They fused together to form a mass 40 mm in its longest diameter. Two subsequent virus treatments with 1-week interval have cleared the secondary growth. Three additional weekly treatments were applied to secure the effect. Each treatment included administration of 1 ml of viral preparation (10^7^ EID50/ml). Three years after the treatment the dog remains alive, without any signs of disease recurrence.

### Case 3

**Figure 2:**
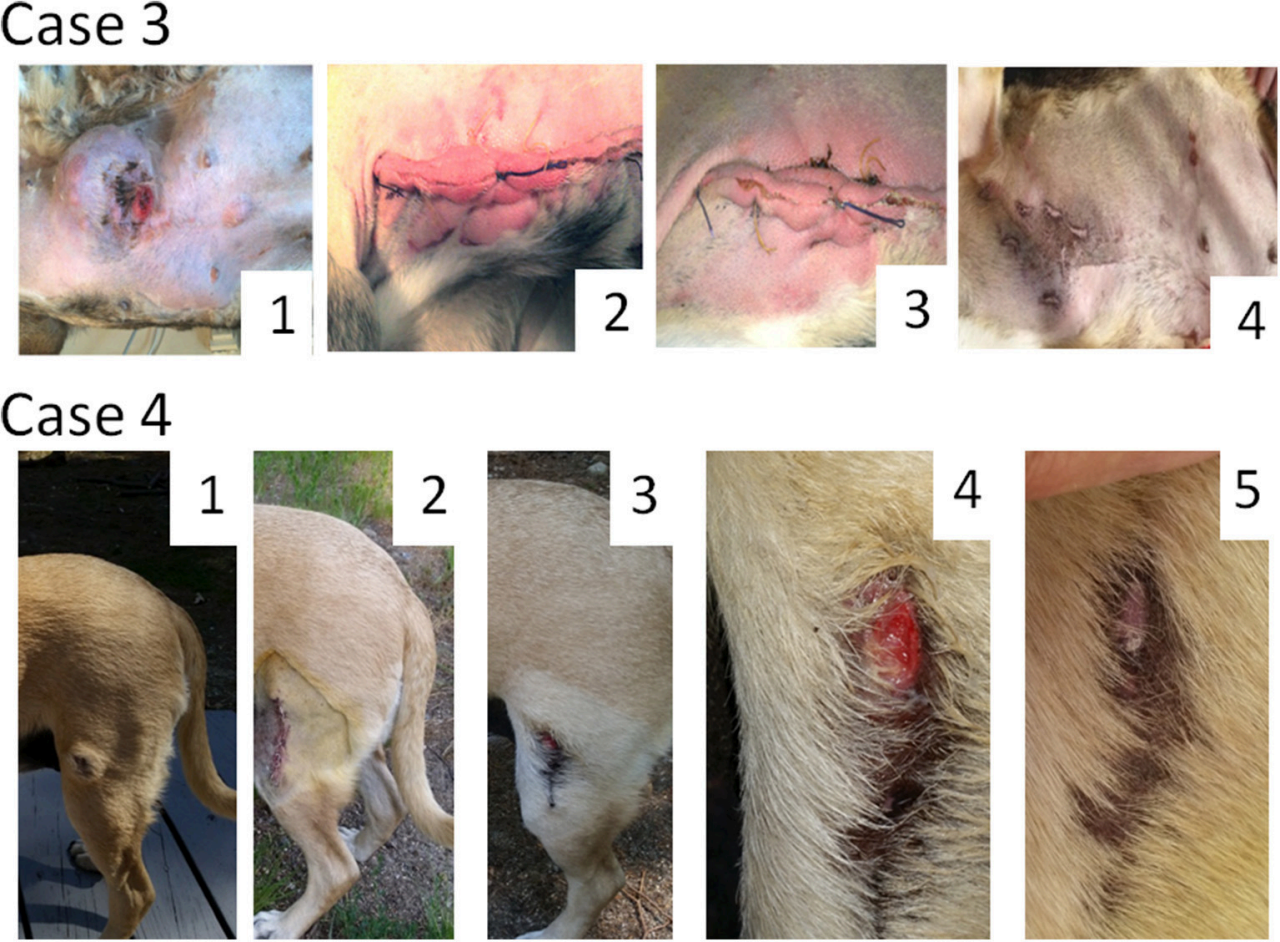
Oncolytic Sendai virus MCT treatments results (Cases 3 and 4). **Case 3**. Male dog of mixed breed of 10 years old was presented with subcutaneous, distant (stage 4) poorly differentiated MCT located on abdomen with multiple palpable metastases. A debulking surgery has been performed. (1) Primary tumor before surgery (120 mm in diameter); (2) MCT secondary growth along the surgical scar, 4 days after the surgery; (3) MCT site, 1 week after the first viral treatment; (4) MCT site, 4 weeks after the first virus treatment. **Case 4**. Female dog of mixed breed of 8.5 years old was presented with cutaneous, local (stage 1) intermediately differentiated MCT. (1) Primary tumor (35 mm in diameter) before any treatment; (2) MCT site after complete surgical removal with at least 5 mm clean margins; (3) MCT relapse, 6 weeks after the surgery; (4) MCT site, 1 week after the first viral treatment; (5) MCT site, 2 weeks after the first virus treatment.

Male dog of mixed breed, 10 years old, presented with subcutaneous MCT in the abdominal region (Figure 2, **Case 3**). Multiple palpable lymph nodes and cutaneous metastases were also detected. According to the owner the primary tumor grew fast, from 30 to 120 mm during a 3-week period. Through fine-needle aspiration the primary tumor and some tested metastases were graded as poorly differentiated. The tumor stage was assigned as “distant” or “stage 4.” Since the radical surgical excision of all metastases was not possible the owner requested a debulking surgery, which has been performed. Histological examination of the excised tumor mass revealed a mitotic index of 9 (10 × HPF) and confirmed the tumor grade. As expected, multiple MCT lesions likely of the same tumor grade (tested by fine-needle aspiration) appeared at the surgical site. Nevertheless, three weekly Sendai virus treatments cleared completely all tumor nodules located at the site of surgical scar. Each treatment included administration of 1 ml of the virus with a titer of 10^7^ EID50/ml delivered through multiple 0.01–0.1 ml injections directly and around the tumor as well as under the tumor bed. Additional 1 ml of viral material with the same titer in 0.01–0.1 ml doses was injected into visible metastatic nodules during each treatment. In total eight weekly applications of the virus were delivered in an attempt to clear metastases. However, even though some metastatic nodules shrank after the treatments, additional nodules continued to appear and grow. The dog was finally euthanized, 2 weeks after the last administration of the virus. Of note, in an attempt to improve quality of life the dog was treated with regular daily oral prednisone (1 mg/kg) 28 days before euthanasia.

### Case 4

Female dog of mixed breed, 8.5 years old, presented with a cutaneous mass of 30 mm in diameter at the left thigh (Figure 2, **Case 4**). Fine-needle aspiration revealed intermediately differentiated MCT. The tumor stage was assigned as likely “localized” or “stage 1” but regional lymph-nodes were not tested. MCT was subjected to virotherapy by administration of 10 weekly virus applications. The therapy did not clear the tumor mass, but the tumor stopped growing and didn't change its size during the treatment period. After the first round of virotherapy the tumor was surgically removed with curative intent and subjected to a histological examination that confirmed the initial diagnosis and revealed the presence of at least 5 mm clean surgical margins. The mitotic index of malignant cells was found to be equal to 0 (10 × HPF). Despite this low mitotic index, localized stage of MCT and comparatively large clean surgical margins, unexpected tumor relapse (tested by fine-needle aspiration) was noticed 6 weeks after the surgery. Nevertheless, resumed virus therapy completely cleared the recurrent tumor mass with only two weekly administrations of Sendai virus. Each treatment included 1 ml of the virus (10^8.6^ EID50/ml). One and a half years after the treatment the dog is live and does not have any signs of disease recurrence.

### Case 5

**Figure 3:**
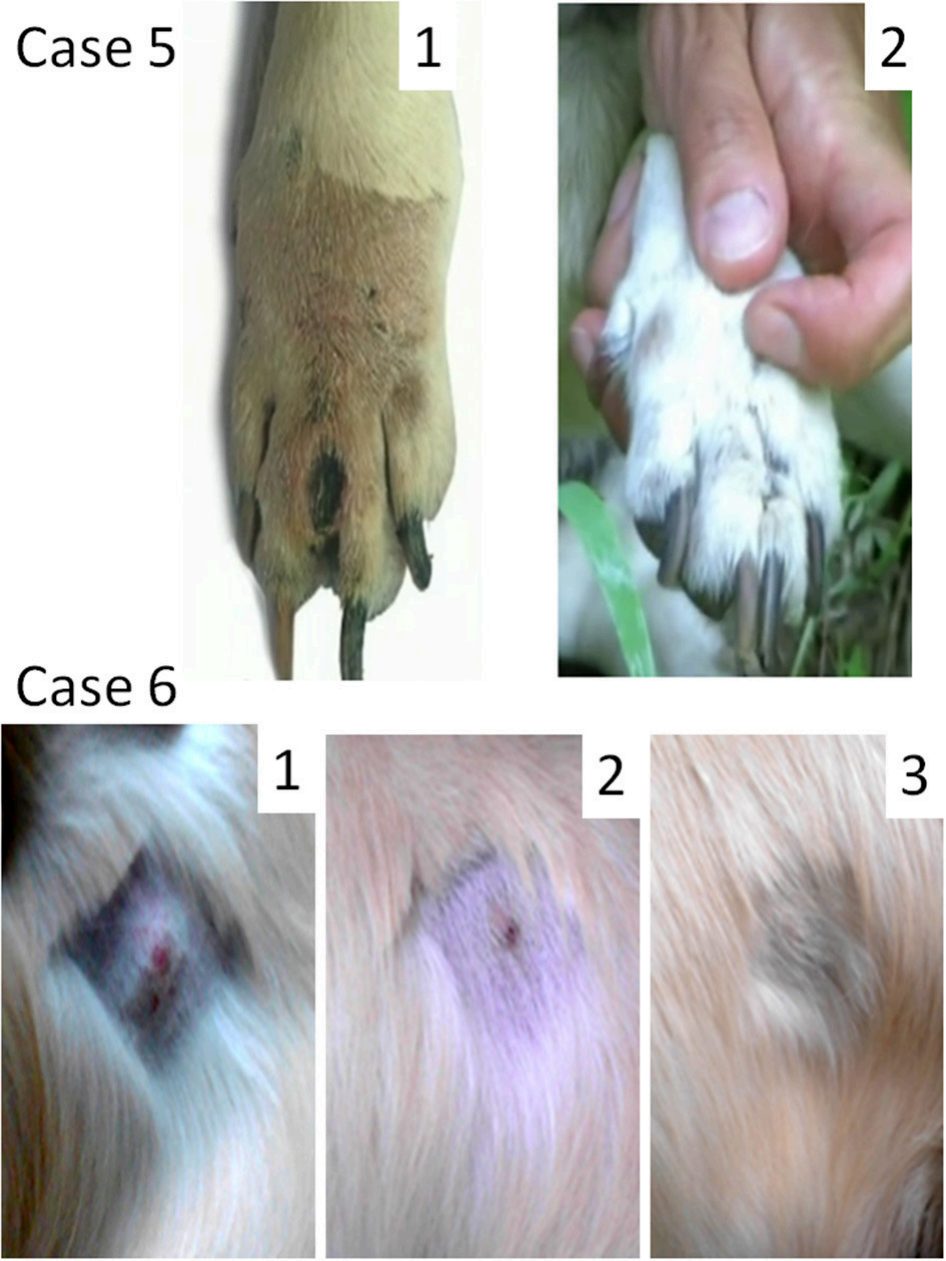
Oncolytic Sendai virus MCT treatments results (Cases 5 and 6). **Case 5**. Female dog of mixed breed of 13 years old was presented with cutaneous intermediately differentiated inter-digital mass of 15 mm. The disease stage was not assigned. (1) Inter-digital MCT, 1 week after prednisone injection and before a surgery; (2) MCT site after debulking surgery and 1 month after final virus treatment. **Case 6**. Male golden retriever of 3 years old was presented with cutaneous well differentiated MCT (20 mm in diameter) in the abdominal region. The disease stage was not assigned. (1) MCT (mildly inflamed with ulceration at its center) before the viral treatment; (2) MCT site 2 weeks after the first viral treatment; (3) MCT site 6 weeks after the first viral treatment.

Female dog of mixed breed, 13 years old, presented with a cutaneous interdigital mass of 15 mm in diameter (Figure 3, **Case 5**). Fine-needle aspiration revealed intermediately differentiated MCT. The tumor stage was not assigned because local lymph nodes were not probed. A radical curative surgery with clean margins was impossible because of the tumor location. A total resection of the foreleg was suggested, but rejected by the owner. MCT was subjected to Sendai virus therapy by administration of 12 weekly applications. The tumor was not cleared but remained the same in size during the virus treatment and 6 months thereafter. Secondary fine-needle aspiration was performed after the end of virus treatment. It confirmed the initial diagnosis as intermediately differentiated MCT, but revealed extensive necrotic areas in the analyzed sample. Nine months after initial diagnosis, a small trauma at the tumor site caused a painful degranulation event, which was treated with one prednisone injection (2 mg/kg). One week after the prednisone injection the tumor was surgically debulked (~3/4 of the mass was removed). Two weeks after the debulking surgery two consecutive weekly 1 ml applications of the virus (10^7^ EID50/ml) into the residual part of the tumor were performed. The remaining tumor mass ultimately regressed. The dog remained tumor free and died from unrelated causes 2 years later. Autopsy did not reveal MCT cells at the former tumor location site.

### Case 6

Male golden retriever, 3 years old, presented with a cutaneous tumor mass (20 mm in diameter) in the abdominal region (Figure 3, **Case 6**). The tumor was mildly inflamed with visible ulceration at its center. Fine-needle aspiration revealed highly differentiated MCT. Another highly differentiated MCT (30 mm in diameter), which was located near the stifle, was surgically removed 2 months prior. The stage of the abdominal tumor was not assigned as far as it was not clear if the new growing mass represented an independent primary MCT or a metastasis of the tumor that was already removed. Virotherapy was suggested and Sendai virus was administered with 2-week intervals [1 ml (10^7^ EID50/ml)]. The tumor was completely cleared after three virus administrations (Figure 3, **Case 6**). Nevertheless, two additional applications were performed to secure the effect. One and half year after the treatment the dog remains alive without any signs of disease recurrence.

## Discussion

In this section we discuss the potential mechanism of Sendai virus oncolytic action, reasons for variable responses to the therapy, viral preparation, virus dosage, side effects, and a possible approach to their prevention.

### Potential mechanism of action

All tested canine patients responded to the viral treatment completely or partially. What mechanism enabled the response? Sendai virus could specifically destroy malignant cells via a number of mechanisms outlined in two recent reviews (66, 67) and briefly summarized here.

#### Direct destruction of cancer cells

One of the possible mechanisms of Sendai virus anticancer activity involves a selective direct destruction of cancer cells. This destruction is most likely promoted by higher affinity of Sendai virus to malignant rather than to normal cells and by the ability of Sendai virus to form syncytia.

Sialic acid polymers are cellular receptors for some paramyxoviruses including Sendai virus (68, 69). The virus has high affinity for its receptor. This high affinity toward sialic acid polymers promotes better binding of Sendai virus to malignant rather than to normal cells because density of sialic acid polymers on the cellular surface correlates with cellular malignancy (70–74).

Sendai virus, like other Paramyxoviruses, is able to spread through formation of large multinucleated structures. By inducing fusion of infected and uninfected cells the virus efficiently spreads without being exposed to neutralizing antibodies. The cell fusion structure, the syncytium, has a survival time of <5 days (75). A tumor cell, which is fused into a syncytium, cannot survive longer.

#### Unmasking cancer antigens

Malignant cells also could be destroyed through specific anti-tumor immunity triggered by Sendai virus. It could happen through unmasking cancer antigens, stimulation of natural killer (NK) and dendritic cells, and up-regulation of interferons and other cytokines. A high level of sialylation of cell membranes correlates with malignancy. Both invasive and metastatic abilities of tumor cells are associated with density of sialic acid polymers on their surfaces (70–74). Viral neuraminidase promotes desialylation of cell membranes (68, 75). Removal of sialic acids from the surface of tumor cells reduces their malignancy and increases immunogenicity, perhaps by unmasking cancer antigens and increasing tumor cell “visibility” to cytotoxic lymphocytes as well as to NK cells (76, 77). In addition, sialidase-treated and, consequently, desialylated tumor cells better activate NK cells for IFN-gamma production (76).

#### Stimulation of natural killer cells

NK cells represent an important component of the innate immune system. NK cells can destroy virus-infected or malignant cells without prior antigen stimulation (78). A study of UV-inactivated Sendai virus demonstrated that virus-mediated tumor regression is enhanced by NK cells (62).

#### Stimulation of dendritic cells

Dendritic cells (DCs) are specialized antigen-presenting cells that efficiently amplify both innate and acquired immune responses against various pathogens and malignant cells (79). A recombinant Sendai could trigger fast activation and maturation of DCs. Within an hour after *ex vivo* viral treatment DCs reach mature phenotype. Sendai virus-activated DCs dramatically improve the survival of animals affected by highly malignant melanoma (80). In model animals even UV-inactivated Sendai virus triggers strong infiltration of a tumor by DCs (60). Sendai virus activated DCs inoculated into animals prior to a tumor cell challenge prevented neuroblastoma growth and prostate adenocarcinoma metastasis into the lungs (81, 82). Similar results were obtained using DCs activated by different variants of recombinant Sendai virus strains with transplantable carcinomas including colorectal (83), squamous cell (84), hepatic and prostate cancers (59).

#### Stimulation of interferons

The Sendai virus is an efficient trigger of interferon alpha secretion by human peripheral blood leukocytes (85). Even UV-inactivated Sendai virus induces interferon-alpha and interferon-beta production in some tumor cell lines (63).

### Variation of responses to viral treatment and potential mechanism of sendai virus action

This pilot study was not directed to treatment of most advanced stages of MCT. Among six dogs only one had confirmed distant metastases and the treatment with Sendai virus although produced positive response, was not curative for the dog. It is not known if the other dogs did or did not have metastases because they were not fully staged.

Despite the rather small sample size, we were able to observe a large spectrum of variable responses to oncolytic virus therapy. In cases 1 and 6 tumor masses were completely cleared without any surgical intervention, after no more than three consecutive virus applications. One of these tumors was poorly differentiated (case 1), while another was well differentiated (case 6). Two tumors of intermediate differentiation (cases 4 and 5) were stabilized only by multiple applications of the virus. However, the virus therapy completely eliminated the secondary tumor growth (case 4) or the tumor mass left after incomplete surgery (case 5). The therapy also completely cleared secondary growth of the intermediately differentiated MCT in case 2, but only partially stabilized the poorly differentiated tumor distant metastases in case 3. The animal in case 3 had most advanced disease (stage 4). Such diversity of responses could have many explanations including individual variability of tumors and their variable sensitivity to the virus, different amount of tumor load, differences in immune status of the dogs etc.

Most likely, malignant cells in the tumors that did not require surgical intervention were highly sensitive to viral infection, while cells in the remaining tumors were more resistant. The fast tumor clearance observed in cases 1 and 6 could be attributed to efficient viral infection and direct lysis of malignant cells. It is likely that the direct lysis was less efficient in cases 4 and 5, because multiple viral treatments were unable to clear the tumors. Such clearance occurred only after surgery at a very early stage of tumor re-growth. We hypothesize that cells most resistant to the virus, could survive virotherapy and resume tumor growth proliferate, particularly in animals with a high tumor load, such as in case 3. However, it is likely that a virus-stimulated immune system is able to clear minimal residual disease if the tumor load is comparatively low.

### Virus preparation

In this study we did not find much difference in the efficiency and manifestations of the two types of virus preparations. Even by using crude virus-containing allantoic fluid there was no significant adverse effects in the animals. Apparently, more clean preparations could be recommended for further studies to avoid potential adverse allergic reactions. The purification scheme for Sendai virus used in the study is relatively inexpensive and simple. However, we did not yet developed a lyophilization protocol for the purified virus, and storage conditions still requires deep freezing at −80°C.

### Treatment dosages

In this work we used two viral treatment dosages with titer 10^7^ or 10^8.6^ EID50 per 1 ml. The lower titer preparation was used in cases 1–3, 5, and 6, and the preparation with the higher titer was used in case 4. The lower-titer virus was able to clear the tumors, while the higher titer virus still did not cause any significant side effects. Therefore we can conclude that either of the doses appears to be acceptable for subsequent studies.

### Side effects

Side effects of the virus treatment were minor and transitory (Table 2). The most bothersome included slight tenderness and edema at the injection sites. Usually, these side effects resolved quickly without any intervention. Nevertheless, it was found that oral administration of diphenhydramine helped in eliminating these problems faster. Perhaps it would be helpful to prevent rather than treat potential pain or edema by using oral diphenhydramine before the virus treatments.

When possible, steroids were avoided. Steroid anti-inflammatory medications are immuno-suppressive and may mitigate the beneficial virus-induced stimulation of antitumor immunity. However, in case 3, prednisone was used as palliative care, after failure of viral treatment.

Allergic reactions to egg products could represent a potential problem because Sendai virus preparations are derived from chicken embryos. We did not notice any allergic reactions in the treated dogs in our pilot study. However, as the possibility exists, a diagnostic skin test for egg allergies could identify vulnerable patients before the onset of virus therapy.

## Conclusions

In summary, we conclude that

- Canine MCTs have different sensitivities to the oncolytic effects of Sendai virus.
- Sendai virus generates a variety of antitumor responses among MCT affected animals, including complete tumor clearance without surgery, complete clearance of reoccurred tumors after surgery, and partial stabilization of the disease.
- Animals with localized disease and small tumor burden have a better chance for complete tumor clearance.
- Side effects of the described oncolytic virotherapy appear minor and transitory.

The Sendai virus mechanism of action does not appear to overlap with any known mechanisms of currently available conventional MCT treatments, thus effective combination protocols of virotherapy with these treatments could be developed. The ability of the virus to clear a remaining tumor after debulking surgery could lead to less intrusive surgical procedures and more frequent preservation of the affected limbs.

The oncolytic Sendai virus therapy of MCTs certainly has limitations; case 3 demonstrated that virus injections might be not helpful against metastases at very advanced stages of the disease. Cases 4 and 5 demonstrated that multiple Sendai virus treatments were only able to stabilize the disease before a surgical intervention. However, our pilot study suggests that Sendai virus can sometimes clear MCT even without surgery (cases 1 and 6); it indicates that the approach is promising, and deserving further studies.

## Ethics statement

This study was carried out in accordance with the recommendations of Guidelines for Institutional Animal Ethics Committees of the Russian Federation. The protocol was approved by the Animal Ethics Committees of Veterinary Clinic of Herzen Oncology Research Institute. Consents for experimental procedures were received from all animals owners.

## Author contributions

GI and EM contributed equally to this work. GI was responsible for designing and supervising of clinical studies with dogs. EM performed all veterinary treatments for dogs. AS manufactured preparations of Sendai virus, performed virus titrations in chicken embryos. OM participated in designing experiments, compiling results, and writing the manuscript. PC initiated and coordinated the study and revised the manuscript.

### Conflict of interest statement

The study was supported by Ministry of Science and Education of Russia, project code RFMEFI60714X0014 and by SATOR Therapeutics BioEnterprise. The funders had no a role in study design, data collection and analysis, decision to publish, or preparation of the manuscript. All the authors declare no other conflict of interest.
